# Testing If Primal World Beliefs Reflect Experiences—Or at Least Some Experiences Identified *ad hoc*

**DOI:** 10.3389/fpsyg.2020.01145

**Published:** 2020-06-24

**Authors:** Jeremy D. W. Clifton

**Affiliations:** Seligman Lab, Positive Psychology Center, Department of Psychology, University of Pennsylvania, Philadelphia, PA, United States

**Keywords:** experiences, primal world beliefs, trauma, socio-economic status, family income, gender, crime

## Abstract

Do negative primal world beliefs reflect experiences such as trauma, crime, or low socio-economic status? Clifton and colleagues recently suggested that primals—defined as beliefs about the general character of the world as a whole, such as the belief that the world is safe (vs. dangerous) and abundant (vs. barren)—may shape many of the most-studied variables in psychology. Yet researchers do not yet know why individuals adopt their primals nor the role of experience in shaping primals. Many theories can be called *retrospective theories*; these theories suggest that past experiences lead to the adoption of primals that reflect those experiences. For example, trauma increases the belief that the world is dangerous and growing up poor increases the belief that the world is barren. Alternatively, *interpretive theories* hold that primals function primarily as lenses on experiences while being themselves largely unaffected by them. This article identifies twelve empirical tests where each theory makes different predictions and hypothesizes that retrospective theories are typically less accurate than interpretive theories. I end noting that, even if retrospective theories are typically inaccurate, that does not imply experiences do not shape primals. I end by offering a conceptual architecture—the Cube Framework—for exploring the full range of human experience and suggest that, though psychologists have historically focused on negative, externally imposed experiences of short-duration (e.g., trauma), positive, internally driven, and longer-term experiences are also worth considering.

## Introduction

After psychologists introduce new constructs, such as learned helplessness or grit (Abramson et al., 1978; Duckworth et al., 2007), many researchers eventually ask an important question: *Which experiences influence (or are influenced by) my construct?* Having recently introduced a construct (Clifton et al., 2019), I turned to this question, beginning with a literature search for a tool that would enable systematic theorizing about a broad range of experiences in relation to my construct. What I found instead were a few organizing frameworks unsuited to this particular task of general theorizing (e.g., Duerden et al., 2018) and a handful of largely overlapping clinically oriented checklists dominated by a particular type of involuntary, negative experiences of quick duration, such as injury or death of a family member (e.g., the Social Readjustment Rating Scale by Holmes and Rahe, 1967; the Life Experiences Survey by Sarason et al., 1978). Moreover, despite positive psychology’s promising departure from psychology’s historical focus on negative experiences (Seligman and Csikszentmihalyi, 2000), the positive psychology literature has yet to produce commensurate checklists of positive experiences. Thus, absent the tool I sought, I conducted the sort of *ad hoc* process that is common among researchers. In this process, hypotheses emerge concerning those experiences the researcher happens to think of, often ones already examined in relevant literatures or ones intersecting personal experience. This process has weaknesses. Chief among them is that research programs can never support a reasonably adequate understanding of the role of experience if no reasonably comprehensive range of *things one personally encounters, undergoes, or lives through*—Merriam-Webster’s definition of *experiences*—is ever considered. Thus, after discussing a newly introduced construct and engaging in a typical process of *ad hoc* literature-driven hypothesis generation, I conclude this article with an atypical offering: a simple yet comprehensive conceptual framework for considering the full range of human experiences called the *Cube Framework*.

## The New(Ish) Construct: Primal World Beliefs

**FIGURE 1:**
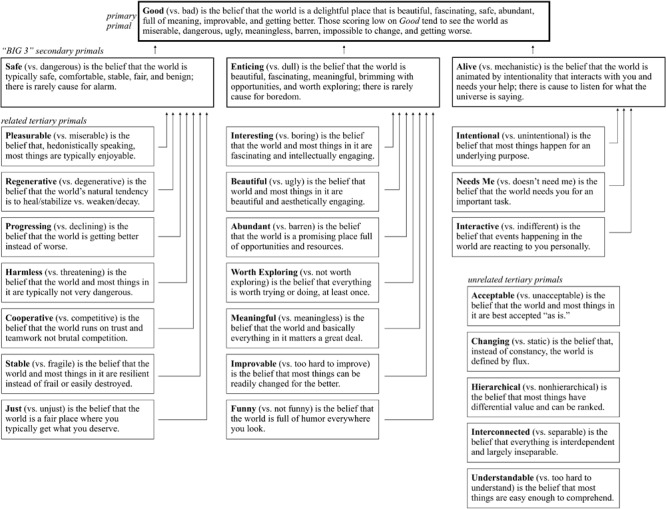
Definitions and structure of the 26 primal world beliefs (22 tertiary, three secondary, and one primary) as identified by Clifton et al. (2019). Reproduced from Clifton and Kim (2020).

For decades various literatures have independently examined the possibility that particular dependent variables, such as political ideology and recovery from trauma, may stem from individual differences in generalized beliefs about the sort of world this is (Janoff-Bulman, 1989; Perry et al., 2013). The most studied is belief in a *Just* world, which is the belief that the world is a place where one gets what one deserves and deserves what one gets. Originally identified by Lerner (1965, 1980) to study the roots of blame and racism, *Just* has since been tied to dozens of variables that *Just* is thought to causally influence. In sum, those higher in *Just* tend to be kinder (presumably because the world rewards kindness); more hard-working (presumably because the world rewards hard work); more successful (because they’ve worked harder, were nicer, and are motivated to *post hoc* justify success); and blame victims like the sick and poor (presumably because they probably got what they deserved). Clifton et al. (2019) recently pulled these literatures together, calling beliefs about the basic character of the world *primals* or *primal world beliefs*, and engaged in an extensive empirical process to map all major primals. We found that *Just* was one of 26 different primals most of which had never been studied (see Figure 1), and many of the new primals are more predictive of human behavior than *Just*, such as the belief that the world is *Beautiful* (vs. ugly) and *Pleasurable* (vs. miserable).

This suggests the plausibility of a truly remarkable scenario described by Clifton and Kim (2020, p. 1). In sum, understanding the behavior of any creature requires observations of that creature in multiple environments. But humans can only ever observe each other in one environment: the world. Not realizing we profoundly disagree about this world along many dimensions, human efforts to understand each other’s behavior should lead inevitably to a specific type of failure: overexaggerating the importance of dispositional differences (i.e., the fundamental attribution error). Thus, it is theoretically possible that psychologists have overlooked a major source of variation in most of the most-studied variables in psychology. Clifton et al. (2019) identify dozens of variables, such as BIG 5 personality traits and subjective well-being, that are likely impacted.

As research exploring the causal role of primals continues, it is worth asking a related but separate question: Where do primals come from? Specifically, which experiences shape (and are shaped by) primal world beliefs? The former question is broad and requires, among other things, a deep discussion of genetics and the ontology of personality traits, which is out of scope. This article concerns the more specific latter question about identifying relevant experiences.

## Retrospective and Interpretive Theories of How Primals Relate to Experiences

Theories of how experiences shape primal world beliefs often fall into two broad types: *retrospective theories* and *interpretive theories*. Retrospective theories suggest that experiences play a key role in shaping primals such that primals often reflect the content of the individual’s background. In this view, for example, the rich are likely to see the world as more *Abundant*, the poor are likely to see the world as more barren (i.e., low *Abundant* scores), and experiencing dangerous environments locally should cause one to see the world as more dangerous globally. This is consistent with an intuitively appealing theory animating much of the pre-existing literature on primals originally posed by traumatologist Janoff-Bulman (1989) and adopted by several others (Foa and Rothbaum, 1998; Foa et al., 1999; Kauffman, 2002; Boelen et al., 2006). This theory holds that traumatic events dramatically increases the belief that the world is dangerous (i.e., low *Safe* scores on the Primals Inventory). Since our (Clifton et al., 2019) identification of several previously unidentified primals, I have observed anecdotally at talks and conferences that similar retrospective intuitions emerge to explain primals’ origins. For example, many researchers intuit that the rich will see the world as a *Good* place and privileged racial majorities will see the world as more *Just* and *Abundant* than minorities. What all these retrospective theories and intuitions have in common is the notion that past experiences characterized by *X* quality pushes the individual toward seeing the world as characterized by *X* quality to such an extent that the individual’s primals reveal not just one’s beliefs but also one’s demographics.

Interpretive theories posit that, rather than a mirror reflecting one’s experiences, a primal functions as a lens used to interpret experiences while being itself largely uninfluenced by them. For example, an interpretive theory of how the primal *Abundant* relates to personal wealth would predict that being rich (or poor) would have little to no impact on the belief that the world is *Abundant*. Likewise, experiencing dangerous environments or trauma (or safe environments) would have little to no impact on the belief that the world is *Safe*. Though such interpretive theories are reasonable, it’s fair to say that they are typically not as intuitively appealing as their retrospective counterparts.

Nevertheless, I hypothesize that interpretive theories are generally more accurate than retrospective theories, though likely with some moderate exceptions such as childhood trauma and chronic pain. My rationale stems from the central point of Janoff-Bulman’s (1989) original article, subtitled *Applications of the Schema Construct*, where she suggests that world beliefs likely operate as *schemas*.

Though definitions of *schema* vary (Van der Veer, 2000), the paradigm has been central to belief research for decades (e.g., Beck, 1963, 1964, 1967, 2005; Crum, 2013; Dweck, 2017). The term usually refers to pre-existing mental models about an object used to generate expectations, assist interpretation and memory reconstruction, and guide interaction (e.g., Piaget, 1926; Rumelhart, 1980; Janoff-Bulman, 1989; Bernstein et al., 1991; Brewer, 2000; Nash, 2013). For example, Davis (1991) found that a schema for an *egg* involves at least 45 different modifiers such as *nutritious*, *delicate*, and *laid in nests*.

In addition to introducing the idea of schemas (1926), Piaget (1971) theorized how schemas would typically relate to experiences. When facing evidence of a schema violation, Piaget posits two options—accommodation (revising one’s schema) or assimilation (reinterpreting the new information to minimize its importance)—and assimilation would be overwhelmingly favored. Decades of research confirms this. When facing schema-inconsistent information, individuals tend to ignore it, reject it, reinterpret it, or adopt other rejection-seeking behavior (e.g., Ross et al., 1975; Hastie, 1981; Janoff-Bulman, 1989; Brewer, 2000). As schema’s influence perceptions, the new information will often serve as “evidence” for the veracity of the original schema (e.g., Vernon, 1955; Labianca et al., 2000), thus creating a *self-supporting* feedback loop. In addition to altering percepts directly, a schema’s influence on behavior can also lead to actual outcomes that provide further “evidence” of the original schema, creating a *self-fulfilling* feedback cycle (e.g., Labianca et al., 2000). In this way, schemas contribute to the phenomenon termed *confirmation bias* (e.g., Merton, 1948; Jussim, 1986; Nickerson, 1998).

Though Janoff-Bulman (1989) acknowledged that “the tendency is toward assimilation rather than accommodation,” she thought trauma would be an exception that would reliably and dramatically alter world assumptions, including what we (Clifton et al., 2019) call *primal world beleifs*. Janoff-Bulman (1992) book on trauma was entitled *Shattered Assumptions* and her theory is sometimes called *shattered assumptions theory*. Yet Kaler et al. (2008) found that in only about a quarter of those recently traumatized was there any reliable change in world beliefs and—moreover—these were equally divided between those coming to see the world more negatively and those coming to see the world more positively. Indeed, as Mancini et al. (2011) note, despite the popularity of shattered assumptions theory, there is little evidence much shattering happens. This is partly due to the absence of control groups, but also the smallness of observed effects which, when it is observed at all, is typically small, even among Holocaust survivors (e.g., Prager and Solomon, 1995). Indeed, if those who experienced first-hand the mass systematic internment, deprivation, torture, and slaughter during the Holocaust—arguably one of the most traumatic events in history—do not see the world as that much worse than those who escaped the experience, then retrospective explanations of how negative primals arise probably has less to offer than intuition suggests.

Yet, as Mancini et al. (2011) point out, shattered assumptions theory remains popular among researchers and clinicians—even lay people—likely in part because of its intuitive appeal. Indeed, after encountering similar patterns of retrospective intuitions in connection to newly identified primals, I have come to suspect several biases are at play, including an actor-observer bias wherein individuals tend to condescendingly imagine that other people cannot help but believe the things they do because of their backgrounds while our own primal world beliefs stem from something more objective and clear-eyed (Clifton, in press). Others are on a journey; I have arrived.

It may be that, rather than experiences influencing primals in a straightforward way, individuals use past experiences to justify whatever primal they already hold. For example, if one sees the world as a dangerous place and gets into a car accident, perhaps on average he will eventually frame that experience as evidence of what he knew all along. Likewise, if one sees the world as a safe place and gets into a car accident, perhaps on average she will eventually frame this experience as exceptional, having occurred for local, particular, and temporary reasons. Indeed, because the world is a giant dataset, there is much information that can be garnered in support of any primal. And if primals direct attention and resist assimilation as the schema literature suggests, researchers should expect such garnering to occur, and thus retrospective theories to be generally inaccurate.

Could a theory explaining how experiences relate to primals be both non-retrospective and non-interpretive? Perhaps. However, whereas retrospective theories could be completely false without fundamentally altering current assumptions about primals and their nature, the same is not true of interpretive theories. Fundamental to our (Janoff-Bulman, 1989; Clifton et al., 2019; Clifton and Kim, 2020; Clifton, in press) understanding of primals is the same assumption underlying researcher’s conceptions of beliefs generally (e.g., Beck, 1963, 1964, 1967, 2005; Crum, 2013; Dweck, 2017). Namely, that beliefs influence thought and behavior largely via ambiguity interpretation. If primals were found to exert no influence on the interpretation of one’s personal experiences, then primals are either (a) exclusively symptoms rather than causes of primals’ numerous personality and well-being correlates; (b) primals’ impact on these outcomes is unmediated by interpretation; or (c) primals do influence the interpretation of some new information but, for some reason, not new personal experiences. Given current research, these options seem unlikely.

## Twelve Hypotheses

To determine whether retrospective or interpretive theories are typically more accurate across different primals and different experiences, ideally multiple hypotheses in which each theory makes diverging predictions should be examined. Table 1 specifies twelve hypotheses which were selected according to three criteria.

**TABLE 1:** Alternative retrospective and interpretive predictions of twelve correlational relationships between primals and experiences.

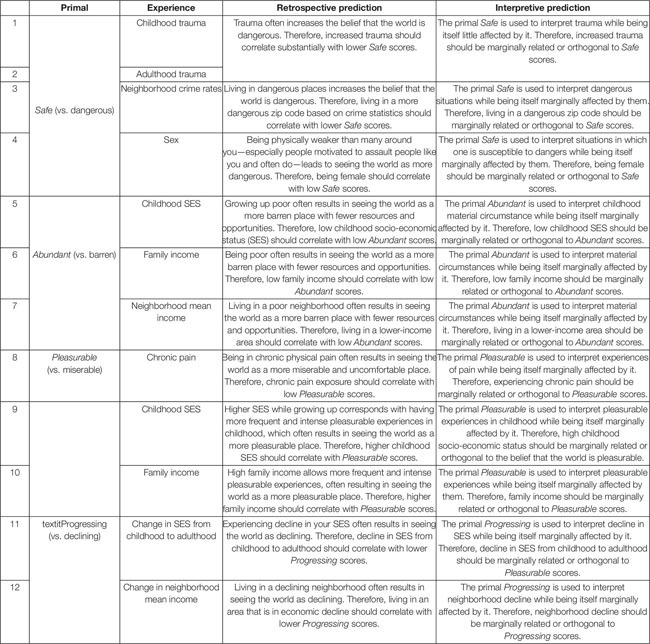

• The measurability of the relevant life experience.
• The involuntariness of the experience (to avoid confounding causal relationships).
• The clarity of alternative retrospective and interpretive predictions.

Multiple hypotheses are necessary because some involve disputable assumptions that others do not. For example, perhaps the most dubious assumption underlies hypotheses #4: Is the world really more dangerous for women than men when men are more likely to be killed violently and die on average 5 years sooner (e.g., Rochelle et al., 2015)? Perhaps, but among a variety of threats that disproportionately impact women, it is indisputable that most women spend life surrounded by biologically stronger, faster, more aggressive individuals who are motivated to assault them, often do, and whose denials are traditionally more likely to be believed over women’s accusations (e.g., Lassek and Gaulin, 2009). Thus, if researchers were to find that nevertheless women and men see the world as equally *Safe*, that can be considered inconsistent with a retrospective theory of how *Safe* develops, though not compelling unless other hypotheses relying on different assumptions are also examined.

All twelve hypotheses can be determined by interpreting correlational effect sizes, with thresholds for interpretation varying depending on the hypotheses. However, based on commonly used thresholds (e.g., Cohen, 1992), the threshold of *r* > 0.30 that Kaler et al. (2008) used to examine a retrospective theory, and my own research experience, I suggest the following admittedly arbitrary thresholds for pairwise relationships:

• *r* > 0.30 can be considered *clearly consistent* with the retrospective prediction and *clearly inconsistent* with the interpretive prediction.
• 0.295 > *r* > 0.20 can be considered *weakly consistent* with the retrospective prediction and *weakly inconsistent* with the interpretative prediction.
• 0.195 > r > 0.10 can be considered *weakly inconsistent* with the retrospective prediction and *weakly consistent* with the interpretive prediction.
• 0.095 > *r* > -0.095 can be considered *clearly inconsistent* with the retrospective prediction and *clearly consistent* with the interpretive prediction.

Because the twelve hypotheses seek to derive conclusions from orthogonality, I would remind the reader that, while correlation does not indicate causation, under certain assumptions orthogonality does suggest causality’s absence or trivialness. Of course, researchers should check those assumptions, particularly curvilinearity, possible third variable confounds, indirect pathways, and counterbalancing effects. For example, Mancini et al. (2016) found that the negative psychological impact of the Virginia Tech shootings was mitigated by the countervailing effects of increased social support which may influence, among other things, beliefs about the world (Mancini, 2019). Nevertheless, if primals do not reflect backgrounds in a straightforward manner as evidenced by bivariate analysis, this would suggest that retrospective theories are inaccurate even if further analysis reveals confounds, indirect pathways, or counterbalancing effects. Retrospective theories are by definition not nuanced in this way.

Previous research sheds light on several of these hypotheses, especially trauma research. For example, converting Prager and Solomon’s (1995) results to a Pearson’s *r* suggests that that subjects who experienced the Holocaust see the world as less benevolent at *r*(158) = 0.31. This is *clearly consistent* with the retrospective prediction and *clearly inconsistent* with the interpretive prediction—but barely so. Using the World Assumptions Scale, Kaler et al. (2008) found in a sample of 735 undergraduates that increased lifetime trauma correlated with world benevolence beliefs at *r* = -0.14 and recent trauma did not seem to have any impact on these beliefs. Given the severity of the Holocaust compared to, say, getting mugged, could it be that *r* = 0.31 approximates an upper-limit trauma effect?

However, because hypotheses concern several primals that only the Primals Inventory measures and because the Primals Inventory is a superior measure of primals (largely by default; for a detailed discussion see Clifton, in press), it is ideal if all twelve hypotheses are examined using the Primals Inventory. To some extent this too has been done. Buried on pages 310–323 of Clifton et al.’s (2019) supplement is a large correlational matrix showing relationships among 524 Americans, ages 18–75 (*M* = 37), who were approximately 50% women and 50% college graduates.

• Concerning Hypothesis #4, women did not see the world as more dangerous than men (*r* = 0.01, *p* > 0.05).
• Concerning Hypothesis #5, growing up poor did not correlate with seeing the world as less *Abundant* (*r* = -0.07, *p* > 0.05).
• Concerning Hypothesis #6, those in families with higher incomes did not see the world as more *Abundant* (*r* = 0.05, *p* > 0.05).
• Concerning Hypothesis #9, growing up poor did not correlate with seeing the world as less *Pleasurable* (*r* = -0.06, *p* > 0.05).
• Concerning Hypothesis #10, high family income did not correlate with seeing the world as more *Pleasurable* (*r* = 0.03, *p* > 0.05).

These results are, based on above thresholds, *clearly inconsistent* with retrospective predictions and *clearly consistent* with interpretive predictions. But these results also come from one sample in which only a preliminary version of the Primals Inventory was used, literally thousands of correlational relationships were examined without correcting for multiple comparisons, above hypotheses were not pre-registered, and most of the twelve hypotheses “were not examined”. Much remains unclear.

## Where Should Researchers Look Instead?

If researchers find that retrospective theories are generally inaccurate, does that mean that experiences do not shape primals? No. Interpretive theories only presume that primals do not reflect the content of past experiences in a straightforward manner, but experiences come in many shapes and sizes and might influence primals in a variety of less straightforward ways. Where could researchers look next? What experiences might researchers focus on?

These questions are impossible to answer without a reasonably exhaustive framework by which a breadth of human experiences can be considered. After recently introducing the primals construct (Clifton et al., 2019), I asked the same question that many researchers before me have asked: *Which experiences influence (or are influenced by) my construct?* Failing to unearth some sort of comprehensive framework or measurement tool that identifies a broad range of psychologically important human experiences that I could use as a basis for systematic theorizing about experiences in relation to my construct, I created the following Cube Framework. I provide it here to aid other researchers examining other constructs, to highlight areas for further research on the primals construct, and to invite comment before using it to build a more comprehensive experience checklist than is currently available.

### Three Dimensions of the Cube Framework

There are three major psychologically salient continuous dimensions by which all experiences vary. For practicality, the Cube Framework simplifies these dimensions into dichotomies. The point is not to know precisely where a particular experience falls on a dimension but for the researcher to have a tool to guard against the consideration of only a narrow slice of human experience.

#### Chronic-Acute

All experiences happen in time. Thus, all experiences can be sorted into more acute experiences that take moments/days/weeks and more chronic experiences that take months/years/decades. Previous experiences checklists have generally ignored chronic life experiences, such as having a chronic illness or negative boss. However, demographic information is often important precisely because it captures chronic experiences, such as being male or poor.

#### Internal-External

All experiences are to varying degrees under the individual’s control. Several literatures draw attention to the psychological importance of this distinction including learned helplessness, attribution theory, optimism/explanatory style, personality, locus of control, and incremental theory (Lewin, 1936; Rotter, 1966; Abramson et al., 1978; Peterson and Seligman, 1984; Blackwell et al., 2007; Harvey et al., 2014). Though many experiences, such as going to college, can be either internally driven or more externally imposed, many experiences can be fairly readily categorized as more often one or the other. A death in the family or inheriting a fortune, for example, are experiences that are usually externally imposed.

#### Positive-Negative

All experiences vary by subjective desirability (good, neutral, or bad). Though most difficult to measure objectively, this dimension is also the most psychologically impactful. There is a massive gulf, after all, between a good childhood and a bad childhood, a good sex life and a bad sex life, and so forth. However, like the internal-external dimension, exactly where any given experience falls on the positive-negative dimension may be up for debate. Nevertheless, many experiences will be readily characterizable. Death and injury, for example, can be thought of as negative. Receiving a promotion or falling in love can be considered positive.

### Eight Types of Experiences in the Cube Framework

**FIGURE 2:**
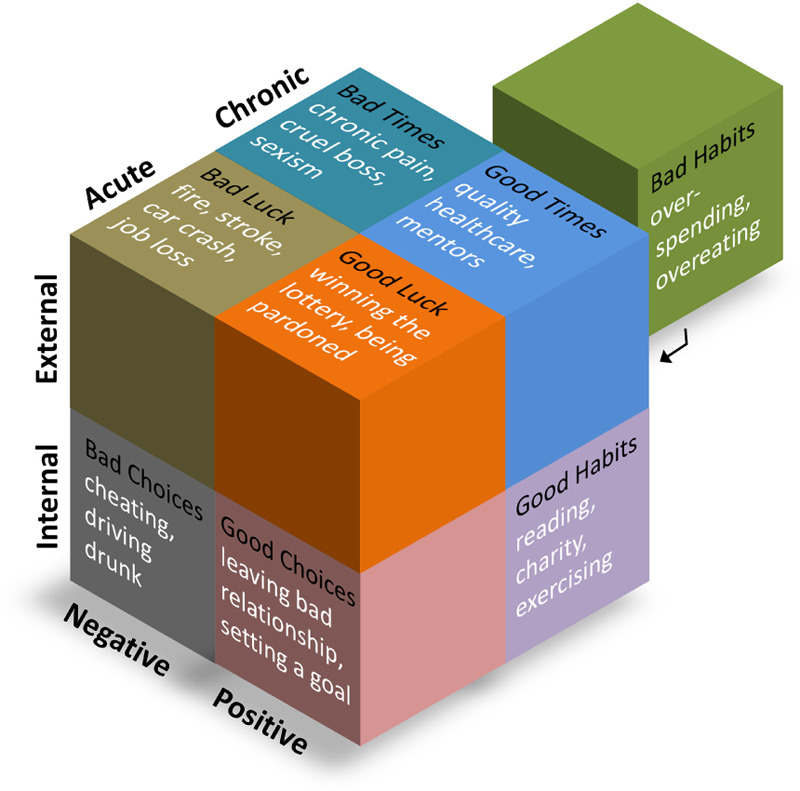
The cube framework uses three dimensions to sort all experiences into eight categories.

The permutations of these three dimensions reveals eight types of human experience (Figure 2).

#### Bad Choices

Acute, internally driven, negative experiences—*bad choices*—may include losing one’s savings in a poor investment, stealing, cheating, sexually assaulting someone, sleeping with a friend’s spouse, deciding to drive home drunk, or joining a cult.

#### Bad Habits

Chronic, internally driven, negative experiences—*bad habits*—may include a gambling habit, smoking, pessimism, distrust, overeating, overspending, continually returning to an abusive partner, or staying in a cult.

#### Bad Luck

Acute, externally imposed, negative experiences—*bad luck*—may include natural disasters, car accidents, stroke, fire, and sudden deaths in the family. The large majority of experiences mentioned by the Social Readjustment Rating Scale (Holmes and Rahe, 1967) and the Life Experiences Survey (Sarason et al., 1978) consists of such *bad luck* experiences. Studying them is worthwhile, but they represent only a narrow slice of life.

#### Bad Times

Chronic, externally imposed, negative experiences—*bad times*—may include being raised by a negative parent, growing up receiving person praise rather than process praise (Kamins and Dweck, 1999); coping with chronic pain, being unemployed, having an unkind boss, involuntarily fighting in a war, or living in a society prejudiced against your gender or race.

#### Good Choices

Acute, internally driven, positive experiences—*good choices*—may include falling in love, identifying your mission in life, taking a backpacking trip across Europe, or converting to a religion.

#### Good Habits

Chronic, internally driven, positive experiences—*good habits*—may include staying physically active, mastering a skill, engaging in some life-giving activity like ballroom dancing or playing in the local philharmonic, chronically believing the best about others, being an avid reader, gardening, spending time outdoors, being in a committed relationship, being an avid traveler, taking care of a dog, volunteering for charity, or raising children.

#### Good Luck

Acute, externally imposed, positive experiences—*good luck*—may include inheriting a fortune, winning the lottery, getting adopted, being recruited for a job, being granted a pardon, or receiving a voucher to go to a better school.

#### Good Times

Chronic, externally imposed, positive experiences—*good times*—may include living in a peaceful society, being raised by a highly supportive parent, receiving a 4-year liberal arts education, enjoying sustained access to medical care, or being mentored by an incredible teacher.

### Using the Cube Framework

Instead of listing out all human experiences, the Cube Framework provides a method that researchers can use to systematically theorize about a diversity of experiences. I suggest using it in two ways. First, the researcher can ask themselves eight questions about each experience type. For example, *What good choices might influence or be influenced by my construct?* However, examining experiences only by type risks the Cube Framework becoming a filter such that only experiences that fit neatly within each type are considered. Addiction, depression, and obesity, for example, are clearly chronic and negative (and important to study) but less clearly categorized along the internal-external dimension, and thus may not emerge from eight questions about the eight types. Therefore, second, I suggest that psychologists also theorize by dimension, one dimension at a time. For example, when considering the acute-chronic dimension I might ask myself: *What experiences that relate my construct might happen in a moment*…*in an hour*…*in a day*…*in a week, in a month*…*in a year*…*in a decade*…*or last a lifetime?* Using both by-type and by-dimension approaches ensures that a diversity of experiences are considered.

The Cube Framework allows flexibility because it is able to incorporate any additional fourth dimension the researcher might deem important. For example, there is arguably at least one other psychologically important dimension on which all experiences vary that the Cube Framework does not incorporate: all experiences can be sorted by the age at which an experience occurs in the life of the person. The Cube Framework does not include this dimension because I found adding it led to the identification of relatively few novel hypotheses, lowered the utility of the framework by complicating it, and, most importantly, age is a characteristic of the person rather than the experience. However, if a researcher wishes to ensure diversity along this or any other fourth dimension, researchers can consider not one cube but two cubes, with each cube labeled according to the fourth dimension, such as *Childhood Experiences* and *Adulthood Experiences*. Then the researcher can consider *childhood bad times* separately from *adulthood bad times, childhood good choices* separately from *adulthood good choices*, and so forth.

### Promising Areas for Further Primals Research

With the big exception of research over the last two decades in positive psychology, psychologists have historically focused on acute, externally imposed, negative (i.e., *bad luck*) experiences like trauma and neglected experiences that last longer, are internally driven, and positive. Thus, when considering which experiences might influence primals, positive and chronic experiences (*good times* and *good habits*), such as having a highly supportive parent or teacher, might be worth further examination. Positive acute experiences, such as powerful moments of transcendence, are also promising.

Furthermore, if retrospective theories are typically inaccurate—if exposure to *X* quality typically has no impact on ways of thinking about the world generally—then perhaps exposure to alternative ways of thinking about *X* quality is what matters. This exposure might occasionally be self-driven by the philosophically adventurous but more typically result from personal social interactions with mentors, friends, colleagues, therapists, parents, or others who see the world differently. Exposure may also occur through storytelling via, for example, movies and novels. For example, a premise of the 2003 and 1999 hit films *Love Actually* and *American Beauty* is that love and beauty are everywhere, even in the midst of pain and suffering—even perversion. Whatever the medium, encounters with alternative lenses on reality may sometimes result in one coming to prefer them. Informal social pressures may also be at work. For example, one unpublished primals research study awaiting duplication indicates that students are more likely than the general public to see the world as dangerous. Is this because the student context is a particularly dangerous one—the retrospective explanation? Likely not. Instead, perhaps the task itself or particular subcultures implicitly encourage—teach—this primal through a variety of formal and informal incentives and social mechanisms. If exposure to different lenses on reality impacts which lenses we choose for ourselves, perhaps researchers will find that one experience that shapes primal world beliefs is taking the Primals Inventory, learning what primals one holds, and discovering one has options.

## Final Remarks

In this article I have asked the typical question a researcher asks after introducing a construct: *Which experiences influence (or are influenced by) my construct?* In the case of primals, I have discussed two broad possibilities. The first holds that primals generally reflect our backgrounds in a fairly straightforward manner (retrospective theories). The second suggests that primals are used to interpret experiences while being themselves marginally influenced by them (interpretive theories). This article has specified twelve empirical tests to determine which approach is typically more accurate, which I hypothesize will most often be interpretive theories despite having less intuitive appeal and running counter to some existing theory. If research confirms this, researchers will have to look elsewhere to determine which experiences might impact primals. To facilitate that search, I have provided the Cube Framework as a tool for methodically considering a range of human experiences and generating hypotheses. My own use of it suggests that a promising place to look will be chronic and positive experiences, such as having a supportive and esteemed parent or mentor who implicitly or explicitly encourages certain primals, as well as acute and positive experiences, such as transcendent experiences.

In closing, however, I confess some pessimism. It may be that few naturally occurring life experiences reliably influence primals. Perhaps primals typically emerge early in life for idiosyncratic reasons in a process non-deterministically yet strongly impacted by genetics. Primals could then perpetuate themselves through mechanisms associated with schemas. This would not mean, however, that primals cannot be changed by experiences, just that they generally are not. Researchers already know that beliefs very similar to primals can be reliably altered through Cognitive Behavioral Therapy (e.g., Beck, 2005). Thus, even if experiences that influence primals cannot be found, perhaps they can be designed.

## Author Contributions

The first and sole author is responsible for all the content of the article.

## Conflict of Interest

The authors declare that the research was conducted in the absence of any commercial or financial relationships that could be construed as a potential conflict of interest.
